# Unveiling the potential of galectin-3 as a diagnostic biomarker for pancreatic cancer: a review

**DOI:** 10.1097/MS9.0000000000001363

**Published:** 2023-10-02

**Authors:** Nicholas Aderinto, Muili O. Abdulbasit, Deji Olatunji, Mariam Edun

**Affiliations:** a Department of Medicine and Surgery, Ladoke Akintola University of Technology. Ogbomoso, Oyo-State; b Department of Medicine and Surgery, University of Ilorin, Ilorin, Kwara State, Nigeria

**Keywords:** biomakers, galectin-3, pancreatic cancer

## Abstract

Early detection of pancreatic cancer is crucial for improving patient outcomes, and identifying reliable biomarkers is a critical research area in this field. Galectin-3 (Gal-3) is a promising candidate for utilisation as a diagnostic biomarker in early-stage pancreatic cancer. This review aims to explore the potential of Gal-3 in pancreatic cancer diagnosis and its implications for precision medicine. Rigorous validation studies are essential to establish the clinical utility of Gal-3, including large-scale investigations to assess its sensitivity, specificity, and predictive value. Combining Gal-3 with existing biomarkers and advanced imaging techniques may enhance the accuracy of early detection. Moreover, Gal-3 holds promise for risk stratification, enabling the identification of high-risk individuals who could benefit from intensified surveillance and early interventions. However, challenges in standardised testing protocols, establishing reference ranges, assay reliability, workflow integration, cost-effectiveness, and healthcare provider education must be addressed for successful implementation. Despite these challenges, Gal-3 presents significant implications for precision medicine in pancreatic cancer management. By unravelling its potential and overcoming the hurdles, Gal-3 could revolutionise early detection, risk stratification, and personalised approaches in pancreatic cancer care. Collaborative efforts and continued research will be crucial in harnessing the full potential of Gal-3 as a diagnostic biomarker for early-stage pancreatic cancer.

## Introduction

Highlights

- By assessing its expression levels or detecting specific alterations, galectin-3 (Gal-3) could provide valuable insights into the presence and progression of pancreatic cancer, enabling early detection and intervention.
- By incorporating Gal-3 into existing diagnostic methods, such as imaging or blood tests, healthcare professionals may achieve more precise and reliable results, leading to timely treatment initiation and improved patient outcomes.
- Understanding the diagnostic significance of Gal-3 in pancreatic cancer can have profound implications for personalised treatment approaches.

Pancreatic cancer, comprising nearly 3% of all cancer cases, is highly lethal, with an estimated 50 550 deaths projected in 2023^1^. Ranking the fourth most prevalent cancer in the United States, it carries one of the lowest 5-year survival rates at just 12%^1^. The increasing incidence of pancreatic cancer over the past 25 years, particularly in North America, Europe, and Australia, has raised concerns regarding public health^2,3^. This rise can be attributed to an ageing population and lifestyle-related risks, including smoking, obesity, diabetes, and alcohol consumption^4^.

**Figure 1:**
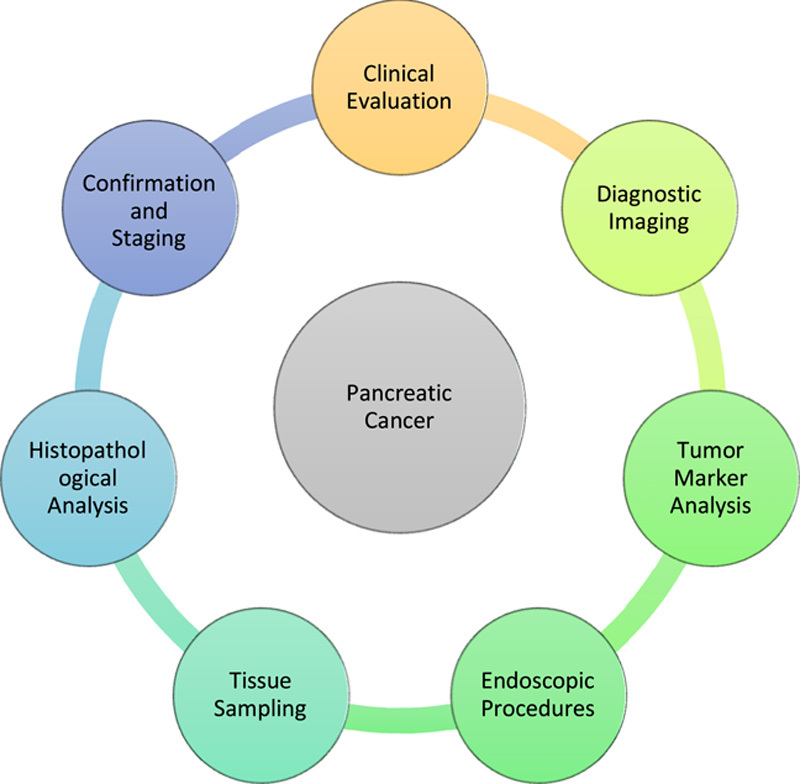
Diagnostic process for pancreatic cancer.

Diagnosing pancreatic cancer is challenging due to non-specific early symptoms, resulting in advanced-stage detection and limited curative treatment options^5^ – Figure 1. Imaging techniques like computed tomography (CT) and magnetic resonance imaging (MRI) play crucial roles in diagnosis, providing detailed imaging of pancreatic abnormalities^6^. Endoscopic ultrasound (EUS) with fine-needle aspiration (FNA) enables safe and minimally invasive sampling^7^. Despite these imaging advancements, early detection remains difficult, necessitating the development of effective diagnostic tools. Galectin-3 (Gal-3) shows promise in this regard.

Gal-3, a chimeric protein located on Chromosome 14, holds promise as a diagnostic biomarker for pancreatic cancer^8^. It plays a role in various biological processes. It exhibits significantly higher expression levels in pancreatic carcinoma tissues and pancreatic cancer patients’ serum than in healthy individuals and those with benign pancreatic conditions^9^. Additionally, different subtypes of pancreatic tumours display distinct patterns of Gal-3 expression, suggesting its potential utility in identifying specific tumour types^10^.

Research has explored Gal-3 as a possible biomarker for pancreatic cancer diagnosis based on serum levels, revealing elevated levels in pancreatic cancer patients^11^. However, it is important to note that Gal-3 alone may not be sufficiently specific for diagnosing pancreatic cancer. Therefore, combining Gal-3 with other established tumour markers, such as carcinoembryonic antigen (CEA) and carbohydrate antigen 19-9 (CA19-9), could enhance the diagnostic sensitivity for pancreatic carcinoma^11^.

This review aims to explore and evaluate the potential of Gal-3 as a diagnostic biomarker for pancreatic cancer. By analysing the current literature and research findings, we seek a deeper understanding of Gal-3’s role in pancreatic cancer diagnosis. This review aims to advance knowledge in pancreatic cancer diagnostics and promote further research and clinical applications of Gal-3 as a valuable biomarker.

## Methodology

A literature search was performed to review the potential of Gal-3 as a diagnostic biomarker for pancreatic cancer. The search strategy involved electronic databases, including PubMed, Scopus, and Web of Science, focusing on articles published between 2000 and April 2023. The following search terms were used: ‘Galectin 3’, ‘pancreatic cancer’, ‘diagnostic biomarker’, ‘prognostic biomarker’, ‘tumour marker’, ‘serum marker’, and ‘biomarker discovery’. Additional relevant articles were identified through manual searches of the reference lists of selected papers. Inclusion criteria encompassed studies that focused on Gal-3 as a diagnostic biomarker for pancreatic cancer. Studies could be original research articles, systematic reviews, meta-analyses, or clinical trials. Exclusion criteria involved studies that did not directly investigate Gal-3 in the context of pancreatic cancer or those that were not written in English. Studies with insufficient data, duplicate publications, or not peer-reviewed were excluded. A narrative synthesis was conducted to summarise and present the findings of the included studies.

## Gal-3: a brief overview

Galectins, a class of glycan-binding proteins, have been studied for their involvement in various biological processes, including cancer development, progression, and metastasis^12^. Gal-3 has garnered significant attention due to its unique structural characteristics and association with fibrosis, inflammation, and cancer^12^. The association of Gal-3 with fibrosis, inflammation, and cancer further underscores its significance in pancreatic cancer pathogenesis^12^. Fibrosis is a hallmark of pancreatic cancer and contributes to tumour progression^11^. Gal-3 has been implicated in regulating fibrosis by promoting the activation of cancer-associated fibroblasts and the deposition of extracellular matrix components^11^. Inflammation is critical in pancreatic cancer development, and Gal-3 has been linked to inflammatory processes^11^. It can modulate the immune response, regulate the secretion of pro-inflammatory cytokines, and contribute to the recruitment of inflammatory cells to the tumour microenvironment^11^. These activities suggest that Gal-3 plays a role in promoting tumour growth and immune evasion.

Gal-3, also known as human Gal-3, is a small lectin with a molecular weight of 26 kDa^13^. It consists of a single carbohydrate recognition domain (CRD) attached to an unstructured N-terminal domain (NTD)^13^. The CRD comprises a β-sandwich structure featuring a 5-stranded β-sheet known as the F-face and a 6-stranded β-sheet that forms a carbohydrate-binding pocket^14^. Gal-3’s CRD recognises and interacts with β-galactoside-containing glycoconjugates, such as lactose and *N*-acetyl-d-lactosamine (LacNAc), through non-covalent interactions^14^. A conserved carbohydrate-binding pocket within the CRD enables Gal-3 to interact with various glycan structures, including extended oligosaccharides like N-glycans and certain glycolipid-type glycans^15^. The reverse side of the CRD, known as the F-face, exhibits a conserved hydrophobic surface that may facilitate the formation of Gal-3 oligomers^16^. Like other lectins, galectins, including Gal-3, play crucial roles as pattern recognition receptors (PRRs) in innate immune responses against pathogens^17^. They recognise various microorganisms such as bacteria, viruses, fungi, and parasites, acting as PRRs similar to soluble molecules like collectins and ficollins, as well as membrane receptors belonging to the C-type lectin family^17^.

Gal-3 plays a critical role in tumour-driven immune suppression^18^. It suppresses the proliferation of T cells with anti-tumour activity, as observed in experiments involving mixed lymphocyte cultures^18,19^. Inhibition of Gal-3 expressed by the tumour leads to the expansion of tumour-reactive T cells. Additionally, Gal-3 secreted by tumour cells can alter macrophage polarisation, promote CD8 T cell apoptosis, and restrict the clustering of T cell receptors (TCRs)^20^. These immunosuppressive effects facilitate tumour escape and contribute to the progression of cancer.

The Gal-3 lattice is a key mechanism by which extracellular Gal-3 influences cellular signalling^21^. This lattice formation modulates cell surface receptor activity by prolonging their retention time or impeding their lateral movement within the plasma membrane^22^. Gal-3 exhibits a range of molecular functions and mechanisms that contribute to its diverse biological activities^22^. One of its key molecular functions is the ability to bind to specific carbohydrate structures through its CRD, facilitating interactions with a wide range of glycan structures^14^. Gal-3 participates in various molecular mechanisms that influence cellular processes through this binding activity^15^.

The formation of protein–glycan complexes is an important mechanism mediated by Gal-3^23^. It can cross-link glycoproteins and glycolipids, forming multivalent complexes that impact cell adhesion, aggregation, and signalling processes^22^. These complexes influence cellular behaviour and communication. Gal-3 is also involved in modulating intracellular signalling pathways^24^. By interacting with intracellular proteins such as kinases, transcription factors, and apoptosis-regulating proteins, Gal-3 can regulate their activity and influence downstream signalling events^23^. This involvement in intracellular signalling pathways contributes to Gal-3’s cell survival, proliferation, and differentiation roles^23^.

Extracellular matrix remodelling is another notable molecular function of Gal-3^25^. It can interact with extracellular matrix components, such as fibronectin and laminin, through its CRD^25^. This interaction influences cell–matrix adhesion, migration, and invasion processes. Gal-3 has been implicated in promoting tumour cell invasion and metastasis through its interactions with the extracellular matrix^21^. Furthermore, Gal-3 participates in immune system regulation^26^. It modulates immune cell functions and regulates inflammatory responses. Gal-3 influences immune cell adhesion, migration, and activation, impacting immune surveillance and immune-mediated processes^26^.

Gal-3 has been the subject of extensive investigation in various types of cancer, focusing on elucidating its role and potential clinical implications^13^. The existing body of research provides valuable insights into the involvement of Gal-3 in different cancer types, shedding light on its molecular functions and potential as a therapeutic target^27^.

Gal-3 has emerged as a significant biomarker in breast cancer, with studies consistently reporting its upregulation in breast cancer tissues compared to normal breast tissues^27,28^. This overexpression has been linked to tumour progression, metastasis, and adverse clinical outcomes^28^. Moreover, Gal-3 has been implicated in promoting cancer cell migration, invasion, and angiogenesis in breast cancer, further emphasising its potential as a key player in disease pathogenesis^28^. Similarly, in colorectal cancer, Gal-3 has been found to exhibit increased expression levels in tumour tissues compared to adjacent normal tissues^29^. This upregulation has been associated with advanced tumour stage, lymph node metastasis, and reduced overall survival rates^29^. These findings highlight the prognostic significance of Gal-3 in colorectal cancer and suggest its potential utility as a predictive marker for disease progression and patient outcomes.

Furthermore, Gal-3 has been investigated in other cancer types, including lung, prostate, ovarian, and gastric cancers. In lung cancer, Gal-3 expression correlates with tumour invasiveness, metastasis, and poor prognosis^30^. In prostate cancer, elevated Gal-3 levels have been associated with aggressive disease characteristics and adverse clinical outcomes^31^. In ovarian cancer, Gal-3 has promoted tumour growth and angiogenesis^32^. In gastric cancer, Gal-3 overexpression has also been linked to advanced disease stages and lymph node metastasis^33^.

The studies exploring the molecular mechanisms underlying the role of Gal-3 in cancer have identified several key pathways and processes influenced by Gal-3. These include modulation of cell adhesion, promoting epithelial–mesenchymal transition (EMT), activating signalling cascades involved in cell survival and proliferation, and regulating immune responses within the tumour microenvironment^34^. Such findings provide a foundation for understanding the multifaceted contributions of Gal-3 to cancer development and progression.

## Gal-3 and pancreatic cancer

Pancreatic cancer presents significant challenges in terms of early detection and diagnosis, necessitating the utilisation of various diagnostic methods^1^. Several approaches are employed in clinical practice to detect and evaluate pancreatic cancer^3^.

Imaging techniques are crucial in the initial detection and staging of pancreatic cancer. CT scans, MRI, and EUS are commonly employed modalities^3^. CT scans provide detailed cross-sectional images of the pancreas, identifying tumour presence and determining its extent^5^. MRI offers enhanced soft tissue visualisation, enabling better characterisation of pancreatic lesions^6^. EUS, which involves using an endoscope with an ultrasound probe, provides high-resolution imaging of the pancreas, facilitating the detection and localisation of tumours^35^.

Tissue biopsy is essential for achieving a definitive diagnosis of pancreatic cancer^36^. Various biopsy techniques are utilised, including FNA guided by imaging modalities such as EUS or CT and surgical biopsies^36^. These procedures involve the collection of small samples of pancreatic tissue for histopathological examination, aiding in the confirmation of malignancy and determination of tumour type^36^.

Blood tests serve as an adjunctive diagnostic tool for pancreatic cancer. CA19-9 is a commonly used blood marker^37^. Elevated levels of CA19-9 can indicate the presence of pancreatic cancer, although its specificity is limited, as it can also be elevated in other benign and malignant conditions^37^. Thus, CA19-9 is often used with other diagnostic methods for comprehensive evaluation.

It is important to note that the choice and combination of diagnostic methods depend on several factors, including the patient’s clinical presentation, imaging findings, and the expertise available at the medical centre^38^. Integration of multiple diagnostic modalities is often necessary to achieve accurate detection, staging, and management of pancreatic cancer^38^. Further research and validation of novel biomarkers hold promise for improving early detection and prognostic evaluation in pancreatic cancer^38^.

## Literature review of Gal-3 in pancreatic cancer

Experimental studies have extensively investigated the role of Gal-3 in pancreatic cancer to elucidate its molecular mechanisms and potential therapeutic implications. These studies have provided valuable insights into the involvement of Gal-3 in various aspects of pancreatic cancer, including tumour progression, metastasis, and therapeutic resistance.

## Prognostic significance and tumour progression

The interaction between tumour cells and pancreatic stellate cells (PSCs) is crucial in tumour progression in pancreatic cancer. According to Zhao *et al*.^39^, Gal-3, significantly expressed in pancreatic tumours, particularly in areas of tumour–PSC interaction, activates PSCs through integrin signalling. This activation leads to the production of cytokines that support tumour growth and immune regulation. Mouse models confirm that tumour–PSC interaction enhances tumour growth and invasion. Interestingly, inhibiting Gal-3 or blocking integrin signalling reduces tumour growth and improves survival. This study underscores the critical role of Gal-3 in mediating tumour–stroma interactions, highlighting its potential as a therapeutic target in pancreatic cancer.

Further research is needed to comprehend the clinical implications of these findings fully. Similarly, Song *et al*.^40^ conducted a study focusing on the upregulation of Gal-3 in human pancreatic tumours and a K-Ras mutant mouse model of pancreatic ductal adenocarcinoma (PDAC). They utilised human pancreatic cancer cell lines and employed various techniques, including protein isolation, immunoblot analysis, and immunohistochemical staining, to assess Gal-3 expression levels. Lentiviral shRNA was used to establish stable cell lines with either overexpressed or silenced Gal-3 expression. The study’s findings shed light on the critical role of Gal-3 in promoting the growth and invasion of pancreatic cancer cells by binding to Ras and activating Ras signalling pathways. These insights into the molecular mechanisms implicated in pancreatic cancer progression may contribute to developing potential therapeutic strategies targeting Gal-3 and Ras signalling in pancreatic cancer.

### Top of form

In a study investigating Gal-3 and its interaction with MUC4 in pancreatic cancer, researchers analysed the serum Gal-3 levels, examined the MUC4–Gal-3 interaction, and assessed its impact on cell adhesion^41^. Significantly higher Gal-3 levels were found in pancreatic cancer patients, particularly those with metastatic disease. Moreover, the interaction between MUC4 and Gal-3 increased pancreatic cancer cell binding to endothelial cells, potentially by clustering MUC4 molecules and exposing adhesion molecules like integrins. Therefore, targeting the MUC4–Gal-3 interaction is a potential therapeutic strategy to prevent pancreatic cancer metastasis by inhibiting cancer cell adhesion to endothelial cells.

In a separate study by Kobayashi *et al*.^42^, the involvement of Gal-3 in the migration and invasion of pancreatic cancer cells was investigated. Gal-3 expression was silenced in pancreatic cancer cell lines using siRNA, resulting in a significant reduction in cell migration and invasion compared to control cells. The study also examined the expression levels of β-catenin, Akt, and GSK-3, which are involved in cancer cell migration and invasion. Notably, Gal-3 silencing reduced the phosphorylation of Akt and GSK-3 and the degradation of β-catenin. These findings suggest that Gal-3 may play a regulatory role in the activity of β-catenin, Akt, and GSK-3 in pancreatic cancer cells. Additionally, the study revealed that control cells exhibited more projecting morphologies than Gal-3-silenced cells, indicating a potential role of Gal-3 in cell shape and cytoskeletal structure.

Gal-3 has been investigated in its interactions with PSCs and pancreatic cancer cells (SW1990 cells). SW1990 cells are a specific cell line derived from human pancreatic adenocarcinoma. The study revealed that Gal-3 promotes the proliferation of SW1990 cells and PSCs, indicating its potential contribution to pancreatic cancer progression by enhancing tumour cell proliferation, invasion, and stromal cell proliferation. These findings significantly impact future therapeutic interventions targeting Gal-3 in pancreatic cancer^43^.

In the study by Liao *et al*., the researchers investigated the role of Gal-3 and S100A9 as potential diabetogenic factors in pancreatic cancer-associated diabetes. They aimed to elucidate the mechanisms through which pancreatic cancer can lead to the development of diabetes. The study involved analysing blood samples from patients with pancreatic cancer-associated diabetes, patients with pancreatic cancer without diabetes, and healthy controls. Gal-3 and S100A9 levels were measured and correlated with clinical parameters, including glucose metabolism and insulin resistance. The results revealed that Gal-3 and S100A9 levels were significantly higher in patients with pancreatic cancer-associated diabetes than in patients without diabetes and healthy controls^44^. Additionally, the levels of these biomarkers were positively correlated with fasting plasma glucose levels and HbA1c, indicating their association with impaired glucose metabolism. Further analysis showed that Gal-3 and S100A9 were involved in promoting insulin resistance. They were found to induce inflammatory responses and impair insulin signalling pathways in pancreatic beta cells and peripheral tissues, leading to insulin resistance and subsequent hyperglycaemia. These findings suggest that Gal-3 and S100A9 may be novel diabetogenic factors in pancreatic cancer-associated diabetes. Their elevated levels in patients with pancreatic cancer-associated diabetes, along with their association with impaired glucose metabolism and insulin resistance, indicate their potential contribution to the development of diabetes in this context. Understanding the mechanisms underlying pancreatic cancer-associated diabetes is crucial for detecting and managing this condition in patients with pancreatic cancer. Gal-3 and S100A9 represent potential targets for therapeutic interventions to prevent or manage diabetes in the context of pancreatic cancer. Further research is needed to explore the therapeutic implications and potential interventions targeting these diabetogenic factors.

In the study conducted by Merlin *et al*.^45^, the researchers investigated the role of Gal-3 in regulating the cellular distribution of MUC1 (mucin 1) and EGFR (epidermal growth factor receptor) in pancreatic cancer cells. They aimed to understand the molecular mechanisms underlying Gal-3, MUC1, and EGFR interaction and its impact on downstream signalling pathways. The study involved pancreatic cancer cell lines manipulated to modulate Gal-3 expression. The researchers analysed the cellular localisation of MUC1 and EGFR in the presence or absence of Gal-3. They also examined the activation of EGFR downstream pathways, including ERK1/2 and Akt. The results revealed that Gal-3 influenced the cellular distribution of MUC1 and EGFR in pancreatic cancer cells. Without Gal-3, MUC1 and EGFR were mainly localised at the cell surface. However, when Gal-3 was present, MUC1 and EGFR were found in intracellular compartments, suggesting that Gal-3 affected their trafficking and subcellular localisation.

Furthermore, the study demonstrated that Gal-3 influenced EGFR downstream signalling pathways. In the presence of Gal-3, the activation of ERK1/2 and Akt was enhanced, indicating that Gal-3 facilitated EGFR-mediated signalling. These findings suggest that Gal-3 plays a regulatory role in pancreatic cancer cells by modulating the cellular distribution of MUC1 and EGFR. This regulation affects downstream signalling pathways, potentially contributing to the progression and aggressiveness of pancreatic cancer^45^.

## Diagnostic significance

In the study conducted by Jiang *et al*.^46^, the researchers investigated the expression of Gal-3 and PTEN (phosphatase and tensin homologue) in PDAC, pancreatic neuroendocrine neoplasms (PNNs), and gastrointestinal tumours using fine-needle aspiration cytology (FNAC). The study aimed to evaluate the potential diagnostic and prognostic value of Gal-3 and PTEN in these tumour types. The researchers collected FNAC samples from patients diagnosed with PDAC, PNNs, and gastrointestinal tumours. They performed immunocytochemistry staining to assess the expression levels of Gal-3 and PTEN in the collected samples. The staining results were then analysed and correlated with the histopathological diagnoses. The study showed that Gal-3 expression was significantly higher in PDAC samples than in PNNs and gastrointestinal tumours^46^. In contrast, PTEN expression was significantly lower in PDAC samples compared to PNNs and gastrointestinal tumours. These differential expression patterns of Gal-3 and PTEN could serve as diagnostic markers to distinguish PDAC from PNNs and gastrointestinal tumours. Additionally, the study found that higher Gal-3 expression was associated with worse overall survival in PDAC patients. This suggests that Gal-3 may have prognostic significance in PDAC, indicating a potential role in predicting patient outcomes. The differential expression of Gal-3 and PTEN observed in PDAC, PNNs, and gastrointestinal tumours on FNAC samples highlights their potential utility as diagnostic markers. The study contributes to understanding these tumours’ molecular characteristics and provides insights into their differential diagnosis and prognosis. Further research and validation studies are warranted to confirm the diagnostic and prognostic value of Gal-3 and PTEN in these tumour types and to explore their underlying mechanisms and potential therapeutic implications.

In the study conducted by Hann *et al*.^47^, a comprehensive analysis of cellular Gal-3 was performed to evaluate its potential oncogenic function in pancreatic cancer cells. The researchers aimed to investigate the role of Gal-3 in pancreatic cancer progression and its potential as a therapeutic target. The study involved the analysis of Gal-3 expression and its functional effects in various pancreatic cancer cell lines. The researchers employed different molecular techniques, including knockdown experiments using siRNA and functional assays to assess cell proliferation, migration, invasion, and apoptosis. Contrary to previous studies suggesting an oncogenic role for Gal-3 in pancreatic cancer, the findings of this study revealed no consistent evidence of Gal-3 acting as an oncogene in pancreatic cancer cells. Knockdown of Gal-3 did not significantly affect cell proliferation, migration, invasion, or apoptosis in the tested cell lines. These results indicate that Gal-3 may not play a major oncogenic function in the context of pancreatic cancer. The study also investigated the potential interaction of Gal-3 with other proteins and signalling pathways involved in pancreatic cancer. The researchers analysed the association of Gal-3 with key proteins, including KRAS, p53, and the EGFR signalling pathway, but found no consistent correlations or significant functional effects.

*In vivo*, studies have also linked Gal-3 to pancreatic tumour growth. A separate study aimed to identify a potential druggable protein target for PDAC^48^. Through liquid chromatography–tandem mass spectrometry (LC-MS/MS), elevated levels of Gal-3 binding protein (Gal-3BP) and progranulin (PGRN) were detected in PDAC compared to breast cancer. Subsequent analysis confirmed the upregulated expression of Gal-3BP in PDAC specimens and plasma samples from patients, with immunohistochemical analysis revealing significant overexpression of Gal-3BP in PDAC compared to normal tissue. Notably, Gal-3BP was found to promote PDAC progression by activating the EGFR-Myc signalling pathway, thereby facilitating tumour growth, migration, invasion, and metastasis. These findings highlight the potential of targeting Gal-3BP with specific antibodies as a promising therapeutic strategy for PDAC treatment.

Gal-3 was examined under hypoxic conditions to assess its expression in pancreatic cancer cells. A study by da Silva Filho *et al*.^49^ investigated the modulation of Gal-3 in pancreatic cancer cells under hypoxia and nutrient deprivation conditions. The researchers observed that Gal-3 expression and secretion were increased in pancreatic cancer cells exposed to hypoxic conditions. Furthermore, nutrient deprivation also led to the upregulation of Gal-3 in these cells. The study suggests that the microenvironmental factors of hypoxia and nutrient deprivation can influence the expression of Gal-3 in pancreatic cancer cells. These findings contribute to our understanding of the molecular mechanisms involved in pancreatic cancer progression and provide insights into the role of Gal-3 as a potential therapeutic target in this disease. In their study, Zhang *et al*.^50^ investigated the role of Gal-3 in the interaction between PSCs and PDAC cells. Human PDAC cell lines and PSCs were cultured with fetal bovine serum, and Gal-3 inhibitors were used. The study aimed to understand the impact of Gal-3 on PSC activation, invasion, migration, and cytokine release. The researchers observed enhanced Gal-3 expression in PDAC cells and tumour-associated stromal cells compared to normal tissues^50^. Furthermore, the study explored the signalling mechanisms underlying Gal-3’s effects on PSCs. Gal-3 treatment stimulated the NF-B signalling pathway in PSCs, leading to increased transcription and production of interleukin-8 (IL-8). The study concluded that Gal-3 activates the ITGB1/ILK signalling pathway, subsequently activating NF-B and upregulating IL-8 expression in PSCs. The study provides insights into the molecular mechanisms underlying the effects of Gal-3 in the tumour microenvironment of pancreatic cancer^51^.

Further investigations explored the interaction of Gal-3 in pancreatic cancer cells with V2-expressing T cells. Gal-3 expression was observed in both PDAC cells and T cells, and the release of Gal-3 by PDAC cells hindered the proliferation of V9V2 T cells. The study also revealed that recombinant Gal-3 suppressed T lymphocyte proliferation, but when Gal-3 levels were reduced in PDAC cells, V9V2 T cell growth was partially restored. These findings shed light on the immunosuppressive mechanisms employed by cancer cells and have implications for developing immunotherapeutic strategies in pancreatic cancer^51^.

In another study, the therapeutic potential of polysaccharide HH1-1, isolated from safflower, was explored in the context of pancreatic cancer^51^. The researchers discovered that Gal-3, which is highly expressed in pancreatic cancer, could be inhibited by HH1-1. Furthermore, HH1-1 modulated the Gal-3/EGFR/AKT/FOXO3 signalling pathway. These results emphasise the therapeutic promise of targeting Gal-3 in pancreatic cancer, with HH1-1 emerging as a promising treatment agent.

According to a study by Gonnermann *et al*.^52^, Gal-3 released by PDAC tumours was found to suppress the proliferation of γδ T cells, a type of T cell involved in the immune response against cancer. However, it did not significantly affect the cytotoxicity of γδ T cells, indicating their ability to kill cancer cells remained intact. The researchers identified the interaction between Gal-3 and specific receptors on γδ T cells as the mechanism underlying the suppression of proliferation. These findings highlight the role of Gal-3 in immune evasion by PDAC tumours and emphasise the importance of understanding the interactions between cancer cells and immune cells for developing effective immunotherapeutic strategies.

In a study by Xie *et al*.^53^, the expressions and clinical significance of tissue and serum Gal-3 in pancreatic carcinoma were investigated. The researchers examined Gal-3 expression in pancreatic carcinoma tissues and serum samples from patients with pancreatic carcinoma. They found that Gal-3 expression was significantly higher in pancreatic carcinoma tissues than in adjacent non-cancerous tissues. Additionally, serum Gal-3 levels were elevated in patients with pancreatic carcinoma compared to healthy controls. The study further analysed the association between Gal-3 expression and clinicopathological parameters in pancreatic carcinoma patients. They observed that high Gal-3 expression in tumour tissues was associated with advanced tumour stage, lymph node metastasis, and poor overall survival.

Similarly, elevated serum Gal-3 levels were correlated with tumour size, lymph node metastasis, and distant metastasis. These findings suggest that Gal-3 expression in both tissue and serum may serve as a potential biomarker for assessing the prognosis and progression of pancreatic carcinoma. Gal-3 could be a valuable indicator for clinical decision-making and monitoring treatment response in patients with pancreatic carcinoma. The study highlights the clinical significance of Gal-3 in pancreatic carcinoma and provides valuable insights for future research and therapeutic interventions^53^.

In a study by Coppin *et al*.^54^, the researchers aimed to evaluate the diagnostic value of CA-125 and Gal-3 in discriminating ductal adenocarcinoma from non-malignant pancreatic diseases. CA-125 and Gal-3 are both biomarkers that have been studied in the context of pancreatic diseases. The study compared the performance of these biomarkers and assessed their potential as diagnostic tools. The results showed that CA-125 exhibited higher diagnostic accuracy than Gal-3 in distinguishing ductal adenocarcinoma from non-malignant pancreatic diseases. CA-125 levels were significantly higher in patients with ductal adenocarcinoma than those with non-malignant pancreatic diseases. On the other hand, Gal-3 levels did not show a significant difference between the two groups. The study concluded that CA-125 and the commonly used biomarker CA19-9 provided better discrimination between ductal adenocarcinoma and non-malignant pancreatic diseases. The combination of CA-125 and CA19-9 improved the diagnostic accuracy, whereas Gal-3 did not contribute significantly to the discrimination. These findings suggest that CA-125, combined with CA19-9, maybe a valuable biomarker panel for distinguishing ductal adenocarcinoma from non-malignant pancreatic diseases. Gal-3, in this study, did not show the same discriminatory capability. The study emphasises the importance of evaluating multiple biomarkers and their combinations to enhance diagnostic accuracy in pancreatic diseases.

## Therapeutic significance

A study by Yi *et al*.^55^ investigated the significance of Gal-3 in pancreatic cancer screening, early diagnosis, prognosis, and therapeutic evaluation. A time-resolved fluorescence immunoassay was performed to assess Gal-3 levels using serum samples from healthy controls and pancreatic cancer patients before and after different treatments. Comparing pre-operative levels, a significant decrease in Gal-3 concentration was observed in patients who underwent radical excision 1 month after surgery. However, patients who underwent palliative resection showed no significant change in Gal-3 levels. Moreover, among patients with radical excision, those with increased or unchanged Gal-3 levels had a significantly higher carcinoma recurrence rate. In a retrospective analysis, Gal-3 exhibited an exceptionally high value and specificity in predicting 3-year survival. Based on these findings, Gal-3 holds potential as a biomarker for pancreatic cancer screening and early diagnosis. Furthermore, Gal-3 may aid in identifying high-risk individuals, monitoring treatment response, and predicting long-term outcomes in pancreatic cancer patients.

In a study by Gaida *et al*.^56^, the expression of Gal-3 in PDAC was investigated. The researchers examined Gal-3 expression in PDAC tissues and compared it to normal pancreatic tissues. They found that Gal-3 expression was significantly increased in PDAC tissues compared to normal tissues. Additionally, they observed that Gal-3 expression was associated with poor prognosis and shorter overall survival in PDAC patients. These findings suggest that Gal-3 may play a role in the development and progression of PDAC. The study highlights Gal-3 as a potential biomarker and therapeutic target for PDAC.

In a study by Shimamura *et al*.^57^, the clinicopathological significance of Gal-3 expression in ductal adenocarcinoma of the pancreas was investigated. The researchers examined Gal-3 expression in PDAC and normal pancreatic tissues. They found that Gal-3 expression was significantly higher in PDAC tissues compared to normal tissues. Furthermore, they observed that high Gal-3 expression was associated with lymph node metastasis and advanced tumour stage in PDAC patients. The study suggests that Gal-3 expression may serve as a useful marker for predicting the aggressiveness and prognosis of PDAC. These findings contribute to our understanding of the clinicopathological implications of Gal-3 in pancreatic cancer.

In the study by Shimura *et al*.^58^, the significance of circulating Gal-3 in patients with pancreaticobiliary cancer was investigated. The researchers aimed to determine the potential of Gal-3 as a diagnostic and prognostic biomarker in this specific type of cancer. The study analysed blood samples from pancreaticobiliary cancer patients and healthy controls. Gal-3 levels were measured and correlated with clinicopathological characteristics, including tumour stage and patient survival. The results demonstrated that circulating Gal-3 levels were significantly higher in pancreaticobiliary cancer patients than in healthy controls.

Moreover, the levels of Gal-3 were associated with tumour progression, with higher levels observed in the advanced stages of the disease. Furthermore, the study evaluated the prognostic value of Gal-3 in patients with pancreaticobiliary cancer. Elevated levels of Gal-3 were correlated with poorer overall survival and disease-free survival rates. This suggests that Gal-3 may serve as a prognostic marker for predicting outcomes in patients with pancreaticobiliary cancer. The findings of this study indicate that circulating Gal-3 holds promise as a diagnostic and prognostic biomarker in pancreaticobiliary cancer. The elevated levels of Gal-3 in patients with this type of cancer and its correlation with tumour progression and poorer survival outcomes suggest its potential clinical significance. Further research and validation studies are warranted to explore the utility of Gal-3 as a biomarker in managing and treating pancreaticobiliary cancer^58^.

These findings highlight the significance of Gal-3 in pancreatic cancer progression and its potential as a therapeutic target. Manipulating Gal-3 expression and interactions may offer novel avenues for developing targeted therapies and immunotherapeutic approaches in managing pancreatic cancer.

## Future directions and implications

Quantifying serum Gal-3 levels in pancreatic cancer patients is commonly performed using the Enzyme-Linked Immunosorbent Assay (ELISA)^59^. This technique employs specific antibodies to capture and detect Gal-3 in patient samples, ensuring high sensitivity and specificity^59^. However, it is important to note that ELISA requires well-equipped laboratories and trained personnel, which may limit its accessibility in certain settings. In the context of detecting Gal-3 expression within pancreatic cancer tissue samples, Immunohistochemistry (IHC) is a prevalent approach^60^. IHC enables the visualisation of Gal-3 under microscopic examination by utilising specific antibodies, providing valuable insights into its cellular localisation and distribution^61^. However, it is worth acknowledging that IHC primarily applies to tissue samples and may not be suitable for routine analysis of serum samples^61^. Western blotting is another widely employed method for assessing Gal-3 expression levels^62^. This technique involves the separation of proteins through gel electrophoresis, followed by their transfer onto a membrane and subsequent antibody-based detection^62^. Western blotting allows for identifying different Gal-3 isoforms and providing semi-quantitative information^63^. Nevertheless, it should be noted that Western blotting requires specialised equipment and expertise, and its application to large-scale serum analysis needs to be expanded^63^.

Emerging technologies, such as multiplex assays, promise to detect Gal-3 expression levels^64^. These assays enable the simultaneous analysis of multiple analytes within a single sample, enhancing efficiency and reducing the required sample volumes^64^. Incorporating Gal-3 within a panel of biomarkers, multiplex assays can potentially improve diagnostic accuracy in pancreatic cancer^65^. However, further research and validation are necessary to establish the clinical utility of multiplex assays in routine practice.

Identifying reliable biomarkers for early detection and screening of pancreatic cancer holds significant potential for improving patient outcomes. Gal-3 has emerged as a promising candidate for diagnostic utilisation in pancreatic cancer screening programmes^6^. However, further exploration is necessary to fully understand its potential applications in this context. Rigorous validation studies are paramount to fully establishing Gal-3 as a diagnostic biomarker for pancreatic cancer. These studies should be conducted on a large scale, encompassing diverse patient populations, to ensure the reliability and generalisability of the findings. By including a wide range of patients with varying demographic characteristics, disease stages, and risk profiles, the sensitivity, specificity, and predictive value of Gal-3 in detecting early-stage pancreatic tumours can be thoroughly assessed. By accurately identifying a higher proportion of true positive cases, Gal-3 demonstrates its potential as an effective biomarker for early detection. It is crucial to determine the sensitivity of Gal-3 in detecting pancreatic tumours at different stages, as early identification significantly improves the chances of successful treatment and improved patient outcomes. Gal-3 should exhibit a high level of specificity to avoid unnecessary diagnostic procedures and potential harm to patients. Validation studies need to include control groups of individuals without pancreatic cancer to assess the specificity of Gal-3 accurately. Accurate determination of these predictive values is essential for making informed clinical decisions and establishing the role of Gal-3 in pancreatic cancer screening programmes.

Robust validation is a prerequisite before integrating Gal-3 into routine screening programmes. Such validation involves multiple independent studies conducted by different research groups to ensure the reproducibility and consistency of the results. The findings should be subjected to rigorous statistical analyses and carefully evaluated for potential confounding factors or biases. Additionally, the performance of Gal-3 should be compared with existing screening methods to assess its incremental value and potential for clinical implementation.

The intricate nature of pancreatic cancer necessitates the exploration of a panel of biomarkers to enhance the accuracy of early detection. Combining Gal-3 with other biomarkers, such as CA19-9, shows promise in improving the sensitivity and specificity of screening tests^66^. Combining multiple biomarkers can improve diagnostic accuracy, as different biomarkers may capture different aspects of the disease^67^.

Among the biomarkers investigated in pancreatic cancer research, CA19-9 emerges as a potential candidate for combination with Gal-3^66^. CA19-9 is currently the most widely used biomarker for pancreatic cancer, and integrating it with Gal-3 may enhance diagnostic accuracy and provide complementary information due to their distinct targets in pancreatic cancer^6^. CEA has also demonstrated utility in various malignancies, including pancreatic cancer^68^. Incorporating Gal-3 with CEA as a multi-marker panel could improve diagnostic specificity and sensitivity^68^.

Furthermore, emerging biomarkers such as microRNAs (miRNAs) hold great potential for enhancing the precision of pancreatic cancer diagnosis^69^. miRNAs, such as miR-21, miR-155, and miR-196a, have been implicated in pancreatic cancer pathogenesis and can be combined with Gal-3 to improve diagnostic accuracy. Moreover, including other biomarkers associated with pancreatic cancer progression and metastasis, such as matrix metalloproteinases (MMPs), may offer valuable insights into disease prognosis and treatment response when combined with Gal-3^70^.

Integrating Gal-3 with advanced imaging techniques, such as EUS or MRI, presents another opportunity to improve the precision and efficiency of pancreatic cancer screening. These imaging modalities provide detailed anatomical information, and by incorporating Gal-3 measurements into the imaging process, the detection of pancreatic tumours may be further enhanced^23^. This integration could enable a more comprehensive disease assessment, combining anatomical and molecular information to improve diagnostic accuracy.

Gal-3 holds promise as a potential biomarker for risk stratification in pancreatic cancer. By assessing Gal-3 levels in individuals with established risk factors, such as a family history of pancreatic cancer or genetic predisposition, it may be possible to identify individuals at a higher risk of developing the disease^38^. This risk stratification approach can potentially guide personalised management strategies for pancreatic cancer, including intensified surveillance or early intervention strategies. Moreover, the inclusion of Gal-3 in risk stratification algorithms has the potential to refine existing risk assessment models. By incorporating Gal-3 measurements alongside established risk factors, such as age, sex, and smoking history, more accurate risk predictions can be made. This can aid in identifying individuals who are at a higher risk of pancreatic cancer, allowing for early intervention and potentially improving patient outcomes.

Continued research efforts and collaboration among multidisciplinary teams are essential to further explore the potential of Gal-3 as a diagnostic biomarker in pancreatic cancer screening programmes. Advancements in biomarker validation, combination strategies, and risk stratification approaches are pivotal in paving the way for the potential integration of Gal-3 into routine screening protocols. These advancements promise to revolutionise early detection and improve outcomes for individuals at risk of developing this aggressive malignancy.

Incorporating Gal-3 testing into clinical practice for pancreatic cancer screening and risk stratification presents various challenges and considerations that must be addressed to ensure its successful implementation. Standardised testing protocols are crucial to ensure consistency and reproducibility of Gal-3 measurements across different laboratories and healthcare settings. Developing comprehensive protocols covering pre-analytical, analytical, and post-analytical phases can minimise variations in sample handling, testing procedures, and result interpretation, leading to reliable and comparable Gal-3 results.

Gal-3’s involvement in numerous cellular processes and its ability to interact with multiple binding partners exemplify its functional significance^13^. However, this versatility poses a challenge in terms of specificity. The lack of specificity in Gal-3 interactions makes attributing specific cellular effects solely to Gal-3 activity challenging. As a result, the interpretation of experimental results becomes complex, and the development of Gal-3-specific therapeutic interventions needs to be improved. Nevertheless, the diverse range of cellular processes that Gal-3 participates in underscores its importance in various biological contexts.

Moreover, the role of Gal-3 in cellular processes and diseases is highly context-dependent. Its effects can be either pro-tumorigenic or anti-tumorigenic, depending on various factors such as the tumour microenvironment, stage of cancer progression, and the presence of other molecular factors^26^. For instance, Gal-3 expression has been associated with poor prognosis in certain cancer types, while in others, it has been linked to better outcomes. Understanding the precise context-specific effects of Gal-3 is essential for accurately targeting it therapeutically and developing effective treatment strategies tailored to specific disease contexts. While Gal-3 levels have been explored as potential biomarkers for various conditions, including cancer and heart failure, their predictive value is limited when considered alone. Due to the complexity and heterogeneity of diseases, Gal-3 levels alone may not provide sufficient diagnostic or prognostic information. However, combining Gal-3 measurements with other clinical markers or developing multi-marker panels may enhance the predictive performance and clinical utility of Gal-3 as a biomarker^59^. Integrating Gal-3 with other clinically relevant parameters may improve the accuracy and reliability of diagnostic and prognostic assessments, thereby facilitating more informed clinical decision-making^60^.

The development of therapeutic interventions specifically targeting Gal-3 presents significant challenges. Gal-3’s multiple binding partners and complex interactions within various cellular pathways make it challenging to achieve selectivity and efficacy in disrupting Gal-3 interactions without interfering with the normal functions of other Galectins^22^. The design and development of therapeutic agents that can selectively modulate Gal-3 activity while minimising off-target effects require careful consideration and extensive research^24^. Additionally, given the diverse roles of Gal-3 in different disease contexts, it is essential to carefully evaluate the potential consequences of modulating Gal-3 activity to avoid unintended effects on disease progression and patient outcomes.

While some trials have demonstrated positive outcomes, such as improved patient survival or therapeutic response, others have not shown significant efficacy^50,51^. These inconsistent findings may be attributed to the complex and multifaceted role of Gal-3 in different disease contexts and the heterogeneity within patient populations. The varying cellular contexts, diverse tumour types, and individual patient characteristics may contribute to the divergent clinical results observed in different studies^51^. Thus, further research, including large-scale clinical investigations involving diverse patient populations, is warranted to elucidate the circumstances in which Gal-3-targeted interventions can yield favourable outcomes.

The cost-effectiveness of Gal-3 testing must be evaluated to determine its feasibility and impact on healthcare resources. Comprehensive cost-effectiveness analyses should consider factors such as the test cost, downstream diagnostic and therapeutic interventions, and potential benefits regarding improved patient outcomes. Understanding the economic implications will inform the decision-making process regarding integrating Gal-3 testing into routine screening programmes and risk stratification algorithms.

Healthcare provider education is vital for successfully incorporating Gal-3 testing into clinical practice. Training programmes and educational resources should be developed to ensure that healthcare professionals know the clinical utility and interpretation of Gal-3 testing. Clear guidelines and algorithms for Gal-3 result interpretation should be provided to support clinical decision-making and risk stratification.

Incorporating Gal-3 testing into pancreatic cancer management reflects the broader implications of precision medicine in improving patient outcomes. Precision medicine aims to tailor medical decisions and interventions to the individual characteristics of each patient, considering factors such as genetic makeup, molecular profiles, and environmental influences^71^. In pancreatic cancer, precision medicine holds tremendous potential to revolutionise treatment strategies and enhance overall management approaches^72^.

Using Gal-3 as a diagnostic biomarker, clinicians can identify individuals at high risk of developing pancreatic cancer. This allows for earlier detection and intervention, potentially improving survival rates. Precision medicine approaches, such as risk stratification based on Gal-3 levels and other biomarkers, enable personalised screening and surveillance strategies for individuals at different levels of risk^73^. This targeted approach optimises the allocation of healthcare resources and reduces unnecessary interventions in low-risk individuals. Moreover, precision medicine in pancreatic cancer management extends beyond risk stratification. Molecular profiling of pancreatic tumours can provide valuable insights into the genetic alterations and molecular pathways involved in each patient’s cancer. This information can guide treatment decisions, allowing for the selection of targeted therapies that address the unique molecular characteristics of the tumour. Gal-3 testing, other biomarkers, and genetic profiling contribute to a comprehensive understanding of tumour biology and inform the development of personalised treatment plans^74^.

The integration of Gal-3 testing and other precision medicine approaches also facilitates the identification of patients likely to benefit from clinical trials and experimental treatments^26^. By selecting patients based on specific biomarker profiles, researchers can enrol individuals more likely to respond positively to novel therapies. This improves the chances of successful outcomes in clinical trials and expedites the development of innovative treatments for pancreatic cancer.

Precision medicine in pancreatic cancer management also has implications for long-term follow-up and survivorship care^75^. By considering individual patient characteristics, such as genetic predisposition and treatment response, clinicians can develop personalised surveillance plans and survivorship programmes tailored to each patient’s needs. This holistic approach addresses the primary tumour and the potential for recurrence, treatment-related side effects, and quality of life issues.

## Conclusion

Detecting tumour biomarkers in blood for pancreatic cancer holds great potential for improving patient outcomes through early detection and risk stratification. Gal-3 has emerged as a promising candidate with implications for these purposes. The future directions and implications of employing Gal-3 as a diagnostic biomarker in pancreatic cancer screening programmes are promising. Large-scale clinical investigations involving diverse patient populations are necessary to assess its sensitivity, specificity, and predictive value. Additionally, combining Gal-3 with existing biomarkers and advanced imaging techniques may enhance accuracy and efficiency in early detection, paving the way for improved patient outcomes.

Moreover, Gal-3 holds significant potential in aiding risk stratification by identifying high-risk individuals who would benefit from intensified surveillance or early intervention strategies. This personalised approach can potentially revolutionise pancreatic cancer management and improve patient outcomes. However, several challenges and considerations must be addressed to incorporate Gal-3 testing into clinical practice successfully. Standardised testing protocols, establishing reference ranges, availability of reliable assays, integration into clinical workflows, cost-effectiveness evaluation, and healthcare provider education are crucial aspects that must be considered.

## Ethical approval

Ethical approval is not applicable for this review.

## Consent

Informed consent is not applicable for this review.

## Sources of funding

No funding was received for this study.

## Author contribution

Conceptualisation: N.A.; writing of the first draft: all authors; writing of the final draft: all authors.

## Conflicts of interest disclosure

All authors declare no conflicts of interest.

## Research registration unique identifying number (UIN)

Not applicable.

## Guarantor

Nicholas Aderinto.

## Data availability statement

No new datasets were generated for this review.

## Provenance and peer review

Not commissioned, externally peer-reviewed.

## Assistance with the study

None.
